# Diabetes association with self‐reported health, resource utilization, and prognosis post‐myocardial infarction

**DOI:** 10.1002/clc.23476

**Published:** 2020-11-04

**Authors:** José C. Nicolau, David Brieger, Ruth Owen, Remo H.M. Furtado, Shaun G. Goodman, Mauricio G. Cohen, Tabassome Simon, Dirk Westermann, Christopher B. Granger, Richard Grieve, Satoshi Yasuda, Jiyan Chen, Katarina Hedman, Carl Mellström, Gunnar Brandrup‐Wognsen, Stuart J. Pocock

**Affiliations:** ^1^ Instituto do Coração (InCor), Hospital das Clinicas da Faculdade de Medicina Universidade de São Paulo São Paulo Brazil; ^2^ Concord Hospital and University of Sydney Sydney Australia; ^3^ London School of Hygiene and Tropical Medicine London UK; ^4^ Hospital Israelita Albert Einstein São Paulo Brazil; ^5^ Terrence Donnelly Heart Centre St Michael's Hospital, University of Toronto Toronto Canada; ^6^ University of Miami Miller School of Medicine Miami Florida USA; ^7^ Department of Clinical Pharmacology and Clinical Research Platform of East of Paris Assistance Publique‐Hopitaux de Paris (APHP) Paris France; ^8^ Sorbonne‐Université (UPMC‐Paris 06) Paris France; ^9^ Department of General and Interventional Cardiology University Heart Center Eppendorf Hamburg Germany; ^10^ German Center for Cardiovascular Research (DZHK), Partner Site Hamburg/Lübeck/Kiel Hamburg Germany; ^11^ Duke Clinical Research Institute Duke University Medical Center Durham North Carolina USA; ^12^ Department of Cardiovascular Medicine National Cerebral and Cardiovascular Center Osaka Japan; ^13^ Guangdong General Hospital, Provincial Key Laboratory of Coronary Disease Guangzhou China; ^14^ AstraZeneca Gothenburg Sweden

**Keywords:** cardiovascular events, diabetes, healthcare resource utilization, myocardial infarction, quality of life

## Abstract

**Background:**

Diabetes mellitus (DM) is associated with increased cardiovascular (CV) risk. We compared health‐related quality of life (HRQoL), healthcare resource utilization (HRU), and clinical outcomes of stable post‐myocardial infarction (MI) patients with and without DM.

**Hypothesis:**

In post‐MI patients, DM is associated with worse HRQoL, increased HRU, and worse clinical outcomes.

**Methods:**

The prospective, observational long‐term risk, clinical management, and healthcare Resource utilization of stable coronary artery disease study obtained data from 8968 patients aged ≥50 years 1 to 3 years post‐MI (369 centers; 25 countries). Patients with ≥1 of the following risk factors were included: age ≥65 years, history of a second MI >1 year before enrollment, multivessel coronary artery disease, creatinine clearance ≥15 and <60 mL/min, and DM treated with medication. Self‐reported health status was assessed at baseline, 1 and 2 years and converted to EQ‐5D scores. The main outcome measures were baseline HRQoL and HRU during follow‐up.

**Results:**

DM at enrollment was 33% (2959 patients, 869 insulin treated). Mean baseline EQ‐5D score (0.86 vs 0.82; *P* < .0001) was higher; mean number of hospitalizations (0.38 vs 0.50, *P* < .0001) and mean length of stay (LoS; 9.3 vs 11.5; *P* = .001) were lower in patients without vs with DM. All‐cause death and the composite of CV death, MI, and stroke were significantly higher in DM patients, with adjusted 2‐year rate ratios of 1.43 (*P* < .01) and 1.55 (*P* < .001), respectively.

**Conclusions:**

Stable post‐MI patients with DM (especially insulin treated) had poorer EQ‐5D scores, higher hospitalization rates and LoS, and worse clinical outcomes vs those without DM. Strategies focusing specifically on this high‐risk population should be developed to improve outcomes.

**Trial registration:**

ClinicalTrials.gov: NCT01866904 (https://clinicaltrials.gov).

## 1 INTRODUCTION

Diabetes mellitus (DM) is a major global epidemic, with a projected 640 million individuals being affected by this disease by 2040.^1^ Moreover, heart disease, including acute myocardial infarction (MI), is not only the leading cause of death but also the leading cause of lost disability‐adjusted life years worldwide. DM is a major factor contributing to this burden of cardiovascular (CV) disease.^2^

It is well established that DM is independently and strongly associated with CV risk.^3^, ^4^ In addition, patients with prior MI who also have DM comprise an important group at a heightened risk of major adverse CV events (MACE), including hospitalizations for heart failure (HF) and all‐cause mortality.^5^, ^6^, ^7^ These associations are even more important given the fact that, even with contemporary standard of care, patients with DM and prior MI have a reduced risk of MACE with prolonged dual antiplatelet therapy,^8^ high‐intensity lipid‐lowering therapy,^9^, ^10^ and newer antihyperglycemic drugs with proven CV outcome benefits.^11^

While the impact of DM in terms of “hard” clinical outcomes, such as recurrent MI, stroke, and CV mortality, is recognized, very little is known regarding its association with patient‐reported outcomes, such as health‐related quality of life (HRQoL) and healthcare resource utilization (HRU).

Several studies have demonstrated that DM may have a considerable negative impact on HRQoL in patients suffering from acute coronary syndrome (ACS),^12^, ^13^, ^14^, ^15^, ^16^ with one report showing no association.^17^ However, these studies had limitations such as small sample size, short‐term follow‐up, or being restricted to a single country.

Data regarding the potential impact of DM on HRU in the long‐term after MI are even more scarce. Janzon et al demonstrated that patients deemed to be at high risk after MI contributed to a substantial burden on HRU.^18^ Considering that DM is highly associated with recurrent hospitalizations due to recurrent ischemic MACE and HF,^5^, ^6^ its potential impact on HRU in a stable post‐MI population is of major interest.

Therefore, we sought to contribute to a better understanding of these issues in patients with DM in the TIGRIS (long‐Term rIsk, clinical manaGement, and healthcare Resource utilization of stable coronary artery dISease) registry, which includes a unique international population with MI 1 to 3 years prior to enrollment.

## 2 METHODS

### 2.1 Study design

TIGRIS was a prospective, observational study that included 9208 patients from 25 countries in Asia‐Pacific/Australia, Europe, North America, and South America. The main objective was to better understand the long‐term outcomes and associated HRU in stable individuals with MI 1 to 3 years prior to study enrollment. A list of principal investigators for the TIGRIS study is provided in Table S1.

TIGRIS (ClinicalTrials.gov, NCT01866904) enrollment criteria as well as patient characteristics and treatment patterns have been published previously.^19^, ^20^ The TIGRIS study was performed in accordance with ethical principles that are consistent with the Declaration of Helsinki, the International Council for Harmonization Good Clinical Practice guidelines, and applicable legislation on nonintervention studies. All participants provided written informed consent. The study protocol and informed consent were reviewed by the corresponding health authorities and ethics boards of all participating study sites. This includes Human Genome Research (HGR) approval in China to include 750 Chinese patients. Patients and the public were not involved in the research process. Briefly, we included patients aged ≥50 years with a documented history of prior MI and at least one of the following risk factors: age ≥65 years, documented history of a second MI >1 year prior to study enrollment, multivessel coronary artery disease, creatinine clearance ≥15 and <60 mL/min, and DM treated with medication. Main exclusion criteria were any condition or circumstance that could limit the complete follow‐up of the patient (eg, end‐stage disease with a life expectancy of <1 year, psychiatric disturbances, alcohol or drug abuse); current participation in a blinded, randomized controlled trial; and treatment with ticagrelor beyond 12 months after MI or off‐label use of ticagrelor.

Data were collected during the initial visit and every 6 months thereafter for 24 months by telephone or in person. The EuroQol Research Foundation survey instrument for measuring self‐reported health status in 5 dimensions (EQ‐5D; mobility, self‐care, usual activities, pain/discomfort, and anxiety/depression) with three levels of severity (EQ‐5D‐3L; none, moderate, and severe) was completed by every individual at each visit.^21^, ^22^

A standardized electronic case report form was used for data collection, which included medical history, demographics, variables from routine examination (at baseline), medication use, HRU, and clinical events (ischemic and hemorrhagic), during follow‐up.

In the present study, 8968 patients (97.2% of the total population) with complete data on DM status were analyzed. Data on self‐reported health status were available in 7260 patients, and data on HRU were available in 7838 patients. The main focus of this study was HRQoL at baseline and HRU during follow‐up.

### 2.2 Statistical analysis

Comparisons of patient characteristics by DM status were summarized by mean and SD for quantitative variables and by frequency/percentage for categorical variables. *P*‐values were based on a 2‐sample *t*‐test, *χ* ^2^ test, and test for trend for quantitative, binary, and ordinal variables, respectively.

For the EQ‐5D‐3L self‐reported health questionnaire, the United Kingdom (UK)‐weighted index has been used as the most widely accepted summary of overall health status.^23^ This combines the five domains (mobility, self‐care, usual activities, pain/discomfort, and depression/anxiety) into a single score. A score of 1 indicates “no problems” on all five items, and a score of less than 1 indicates increasingly poor self‐rated health status, down to a rare score of 0 indicating a state rated as “bad as being dead.”

Hospitalizations and other HRU items were based on 6‐monthly patient recall during follow‐up at 6, 12, 18, and 24 months after enrollment. These have been totaled to summarize each patient's HRU over 24 months of follow‐up.

The 2‐year percentage incidence of clinical outcomes is reported by DM status, using Kaplan‐Meier estimates to account for patients lost to follow‐up. Poisson regression models were used to adjust for other baseline influences on risk of event. Variables included in the adjustment were those in the TIGRIS risk index model,^24^ which were age ≥65 years, second prior MI, chronic kidney disease, HF, peripheral artery disease, CV event in the past 6 months, major bleeding, medical management of index MI, diuretic use, EQ‐5D‐weighted index score, region, and country (as random effects). All analyzes were performed using STATA version 15.1.

## 3 RESULTS

In this population of 8968 patients 1 to 3 years post‐MI, 2959 (33%) had DM, of whom 869 (29%) were treated with insulin (see CONSORT diagram; Figure S1). Baseline characteristics of the population by DM status are described in Table S2. Compared with patients without DM, patients with DM were younger (67.5 vs 65.8 years) and had a higher body mass index (BMI; 26.9 vs 28.3 kg/m^2^). Patients with DM also had a higher prevalence of peripheral artery disease (5.2% vs 9.8%), HF (10.0% vs 14.6%), anemia (2.3% vs 4.1%), angina (8.9% vs 12.2%), and stroke (3.7% vs 5.9%). Moreover, patients with DM also had higher CV event‐related hospitalization rates in the 6 months prior to enrollment (5.0% vs 6.4%). All these measures were more pronounced in patients with insulin‐dependent DM, which comprised both patients with type 1 (N = 86) and type 2 (N = 783) DM (Table S3). Table S4 shows the prevalence of DM by region and country. The highest prevalence of DM was observed in Asia and Australia (37.0%), followed by Latin America (36.3%). The lowest prevalence was observed in Europe (29.2%).

Table S5 shows the utilization of evidence‐based treatments at enrollment by DM status. Of note, the use of dual antiplatelet therapy, angiotensin‐converting enzyme inhibitors/angiotensin receptor blockers, and beta‐blockers was higher in patients with DM. Conversely, patients without DM were treated more often with statins and/or other lipid‐lowering drugs.

**FIGURE 1:**
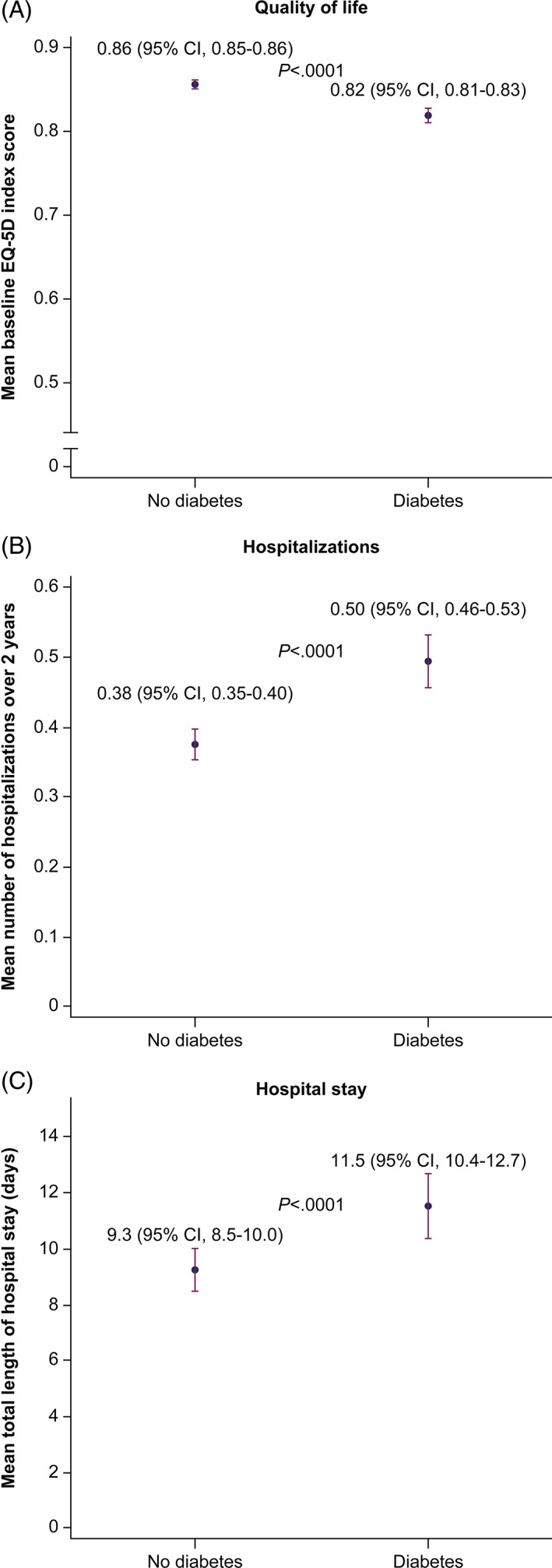
Quality of life and healthcare utilization. A, Quality of life. B, Hospitalizations. C, Hospital stay. Abbreviations: CI, confidence interval; EQ‐5D, EuroQol Research Foundation survey instrument for measuring self‐reported health status in 5 dimensions

Table 1 and Figure 1A show the association of DM with self‐reported health status at baseline using the EQ‐5D‐3L questionnaire. Patients with DM reported more problems in each domain of the EQ‐5D questionnaire, which resulted in an overall significantly worse mean EQ‐5D UK‐weighted index score at enrollment. The mean EQ‐5D visual analog score was also significantly worse in patients with DM. For all these aspects of EQ‐5D, patients with insulin‐treated DM fared worse than patients with noninsulin‐treated DM. Table 2 documents how self‐reported health status was poorer in patients with DM, especially those treated with insulin, and also deteriorated more over the 2‐year follow‐up. These associations persisted after adjustment for other patient characteristics and were driven by significantly poorer scores in each domain of the EQ‐5D questionnaire, with the exception of anxiety/depression in which solely insulin‐treated DM patients fared worse.

**TABLE 1:** Self‐reported health (EQ‐5D) at baseline by diabetes status

	No diabetes	Diabetes	*P*‐value	Noninsulin‐treated diabetes	Insulin‐treated diabetes
	N = 6009	N = 2959		N = 2090	N = 869
EQ‐5D UK‐weighted index score^a^, mean ± SD	0.86 ± 0.20	0.82 ± 0.23	<.0001	0.84 ± 0.21	0.77 ± 0.26
1	3043 ± 50.9	1271 ± 43.2	**–**	962 ± 46.3	309 ± 35.8
0.75‐0.99	1524 ± 25.5	801 ± 27.2	**–**	581 ± 28.0	220 ± 25.5
0.70‐0.74	521 ± 8.7	271 ± 9.2	**–**	178 ± 8.6	93 ± 10.8
0.65‐0.69	337 ± 5.6	200 ± 6.8	**–**	128 ± 6.2	72 ± 8.3
<0.64	553 ± 9.3	398 ± 13.5	**–**	229 ± 11.0	169 ± 19.6
EQ‐5D visual analog scale^b^ (0‐100), mean ± SD	77.0 ± 16.4	74.2 ± 17.8	<.0001	75.4 ± 17.3	71.5 ± 18.7
>92.5	776 ± 13.0	282 ± 9.6	**–**	221 ± 10.7	61 ± 7.1
82.5‐92.5	1390 ± 23.3	660 ± 22.5	**–**	476 ± 23.0	184 ± 21.3
72.5‐82.5	1786 ± 30.0	844 ± 28.8	**–**	626 ± 30.3	218 ± 25.3
62.5‐72.5	1000 ± 16.8	462 ± 15.8	**–**	312 ± 15.1	150 ± 17.4
52.5‐62.5	445 ± 7.5	278 ± 9.5	**–**	186 ± 9.0	92 ± 10.7
42.5‐52.5	360 ± 6.0	239 ± 8.2	**–**	150 ± 7.2	89 ± 10.3
0‐42.5	201 ± 3.4	166 ± 5.7	**–**	98 ± 4.7	68 ± 7.9
EQ‐5D mobility	**–**	**–**	**–**	**–**	**–**
Some problems	1303 (21.8)	877 (29.8)	<.0001	551 (26.5)	326 (37.8)
Severe problems	7 (0.1)	7 (0.2)	**–**	4 (0.2)	3 (0.3)
EQ‐5D self‐care	**–**	**–**	**–**	**–**	**–**
Some problems	269 (4.5)	206 (7.0)	<.0001	117 (5.6)	89 (10.3)
Severe problems	22 (0.4)	17 (0.6)	**–**	8 (0.4)	9 (1.0)
EQ‐5D usual activities	**–**	**–**	**–**	**–**	**–**
Some problems	962 (16.1)	579 (19.7)	<.0001	369 (17.8)	210 (24.3)
Severe problems	45 (0.8)	56 (1.9)	**–**	26 (1.3)	30 (3.5)
EQ‐5D pain/discomfort	**–**	**–**	**–**	**–**	**–**
Some problems	1899 (31.8)	1059 (36.0)	<.0001	713 (34.3)	346 (40.1)
Severe problems	138 (2.3)	105 (3.6)	**–**	53 (2.5)	52 (6.0)
EQ‐5D depression/anxiety	**–**	**–**	**–**	**–**	**–**
Some problems	1222 (20.4)	647 (22.0)	.008	418 (20.1)	229 (26.5)
Severe problems	91 (1.5)	66 (2.2)	**–**	42 (2.0)	24 (2.8)

*Note*: Summary statistics are presented as n (%) unless otherwise stated. For both the scales, higher scores indicate better self‐rated health status.

Abbreviations: EQ‐5D, EuroQol Research Foundation survey instrument for measuring self‐reported health status in 5 dimensions; UK, United Kingdom.

^a^ For the EQ‐5D UK‐weighted index, a score of 1 indicates no problems for mobility, self‐care, usual activities, pain/discomfort, or depression/anxiety, whereas a score of 0 indicates a state as bad as death.

^b^ For the EQ‐5D visual analog scale, a score of 100 indicates “best health you can imagine,” while a score of 0 indicates “worst health you can imagine.”

**TABLE 2:** Association of diabetes (overall and by insulin treatment) with self‐reported health status at baseline and change in self‐reported health status over 2 years' follow‐up

Mean EQ‐5D UK‐weighted index score at baseline (N = 8919)				
	Crude effect^a^ (95% CI)	*P*‐value	Adjusted effect^a^ ^,^ ^b^ (95% CI)	*P*‐value
Diabetes	−0.038 (−0.047 to −0.029)	<.0001^c^	−0.030 (−0.039 to −0.021)	<.0001^c^
Noninsulin‐treated diabetes	−0.018 (−0.028 to −0.007)	<.0001^d^	−0.013 (−0.023 to −0.003)	<.0001^d^
Insulin‐treated diabetes	−0.087 (−0.102 to −0.072)		−0.073 (−0.087 to −0.058)	
EQ‐5D domains at baseline				
	Crude OR^e^ (95% CI)	*P*‐value	Adjusted OR^b^ ^,^ ^e^ (95% CI)	P‐value
Mobility				
Diabetes	1.53 (1.39‐1.69)	<.0001^c^	1.55 (1.39‐1.73)	<.0001^c^
Noninsulin‐treated diabetes	1.30 (1.16‐1.46)	<.0001^d^	1.32 (1.16‐1.50)	<.0001^d^
Insulin‐treated diabetes	2.20 (1.89‐2.56)		2.23 (1.89‐2.63)	
Self‐care				
Diabetes	1.60 (1.34‐1.92)	<.0001^c^	1.53 (1.26‐1.85)	<.0001^c^
Noninsulin‐treated diabetes	1.25 (1.01‐1.55)	<.0001^d^	1.22 (0.97‐1.53)	<.0001^d^
Insulin‐treated diabetes	2.51 (1.97‐3.19)		2.34 (1.80‐3.04)	
Usual activities				
Diabetes	1.37 (1.23‐1.53)	<.0001^c^	1.37 (1.22‐1.55)	<.0001^c^
Noninsulin‐treated diabetes	1.16 (1.02‐1.32)	<.0001^d^	1.18 (1.03‐1.36)	<.0001^d^
Insulin‐treated diabetes	1.95 (1.66‐2.30)		1.90 (1.59‐2.27)	
Pain/discomfort				
Diabetes	1.28 (1.17‐1.40)	<.0001^c^	1.25 (1.14‐1.38)	<.0001^c^
Noninsulin‐treated diabetes	1.13 (1.02‐1.25)	<.0001^d^	1.13 (1.01‐1.26)	<.0001^d^
Insulin‐treated diabetes	1.72 (1.49‐1.99)		1.61 (1.38‐1.88)	
Anxiety/depression				
Diabetes	1.14 (1.03‐1.27)	.01^c^	1.03 (0.92‐1.15)	.65^c^
Noninsulin‐treated diabetes	1.02 (0.90‐1.15)	<.0001^d^	0.91 (0.80‐1.03)	.0002^d^
Insulin‐treated diabetes	1.48 (1.27‐1.74)		1.35 (1.14‐1.60)	
Mean change in EQ‐5D‐weighted index score over 2 years (N = 7260)				
	Crude effect^f^ (95% CI)	*P*‐value	Adjusted effect^b^ (95% CI)	*P*‐value
Diabetes	−0.017 (−0.026 to −0.008)	.0003^a^	−0.016 (−0.025 to −0.007)	.0005^a^
Noninsulin‐treated diabetes	−0.008 (−0.018 to 0.002)	<.0001^d^	−0.008 (−0.018 to 0.002)	<.0001^d^
Insulin‐treated diabetes	−0.040 (−0.055 to −0.025)		−0.038 (−0.052 to −0.023)	
Change in EQ‐5D domains over 2 years				
	Crude OR^g^ (95% CI)	*P*‐value	Adjusted OR^b^ ^,^ ^g^ (95% CI)	*P*‐value
Mobility				
Diabetes	1.27 (1.12‐1.44)	.0002^c^	1.34 (1.17‐1.54)	<.0001^c^
Noninsulin‐treated diabetes	1.12 (0.97‐1.30)	<.0001^d^	1.18 (1.01‐1.38)	<.0001^d^
Insulin‐treated diabetes	1.69 (1.39‐2.05)		1.83 (1.47‐2.27)	
Self‐care				
Diabetes	1.48 (1.18‐1.84)	.0005^c^	1.54 (1.22‐1.94)	.0002^c^
Noninsulin‐treated diabetes	1.31 (1.02‐1.68)	.0002^d^	1.38 (1.06‐1.79)	.0001^d^
Insulin‐treated diabetes	1.91 (1.39‐2.63)		1.99 (1.42‐2.79)	
Usual activities				
Diabetes	1.35 (1.17‐1.57)	<.0001^c^	1.35 (1.16‐1.58)	.0002^c^
Noninsulin‐treated diabetes	1.14 (0.97‐1.36)	<.0001^d^	1.15 (0.96‐1.38)	<.0001^d^
Insulin‐treated diabetes	1.94 (1.56‐2.41)		1.96 (1.55‐2.48)	
Pain/discomfort				
Diabetes	1.19 (1.07‐1.33)	.002^c^	1.18 (1.05‐1.33)	.006^c^
Noninsulin‐treated diabetes	1.08 (0.96‐1.23)	<.0001^d^	1.08 (0.95‐1.24)	.0003^d^
Insulin‐treated diabetes	1.51 (1.27‐1.80)		1.46 (1.21‐1.76)	
Anxiety/depression				
Diabetes	1.13 (0.99‐1.28)	.07^c^	1.04 (0.91‐1.20)	.54^c^
Noninsulin‐treated diabetes	1.01 (0.87‐1.18)	.002^d^	0.95 (0.81‐1.11)	.01^d^
Insulin‐treated diabetes	1.43 (1.17‐1.75)		1.33 (1.07‐1.65)	

Abbreviations: ANCOVA, analysis of covariance; BMI, body mass index; CI, confidence interval; EQ‐5D, EuroQol Research Foundation survey instrument for measuring self‐reported health status in 5 dimensions; OR, odds ratio; UK, United Kingdom.

^a^ Mean effects are for each diabetes group vs non‐diabetes group. Adjusted mean effects use a linear regression model adjusted for age, sex, region, BMI, years in education, and smoking status as fixed effects and country as a random effect.

^b^ Adjusted for age, sex, region, BMI, years in education, smoking status, and baseline EQ‐5D‐weighted index score as fixed effects and country as a random effect.

^c^ Comparison between diabetes vs no diabetes.

^d^ Global test for no association with diabetes group (no diabetes, noninsulin‐treated diabetes, and insulin‐treated diabetes).

^e^ ORs were estimated using ordinal polytomous regression models and the odds of having moderate/severe problems were compared to the odds of having no problems or the odds of having severe problems to the odds of having no/moderate problems in each respective domain.

^f^ Adjusted for EQ‐5D‐weighted index score using ANCOVA.

^g^ ORs were estimated using ordinal polytomous regression models and the odds of having improved were compared to the odds of having no change/worsened over 2 years' follow‐up or the odds of having no change/improved to the odds of having worsened over 2 years' follow‐up in each respective domain.

In terms of HRU, Table 3 and Figure 1B,C shows the incidence and duration of all‐cause hospitalization in the 2 years following enrollment. The percentage of patients hospitalized in the group without DM was lower vs that in the group with DM (24.7% vs 29.9%), with a mean of 0.38 vs 0.50 hospitalizations over the 2‐year follow‐up, respectively. The mean duration of hospitalization was also longer in patients with DM vs those without DM (9.25 vs 11.53 days). Table S6 shows the incidence of hospitalizations for CV events and bleeding events, emergency, and general practitioner visits for CV or bleeding events, cardiologist visits, and other specialist visits. The results are consistent with those shown in Table 1, with higher HRU in those with DM than in those without DM. All these aspects of HRU (except for cardiologist visits) were more pronounced in patients who were treated with insulin.

**TABLE 3:** Hospitalizations for any cause during 2‐year follow‐up by diabetes status^a^

	No diabetes	Diabetes	*P*‐value	Noninsulin‐treated diabetes	Insulin‐treated diabetes
	N = 5249	N = 2589		N = 1866	N = 723
Mean number of hospitalizations (95% CI)	0.38 (0.35‐0.40)	0.50 (0.46‐0.53)	–	0.44 (0.40‐0.49)	0.63 (0.55‐0.71)
Number of hospitalizations, n (%)	**–**	**–**	<.0001	–	–
0	3951 (75.3)	1817 (70.2)	–	1354 (72.5)	464 (64.2)
1	889 (16.9)	486 (18.8)	–	339 (18.2)	147 (20.3)
2	259 (4.9)	158 (6.1)	–	98 (5.3)	60 (8.3)
3	93 (1.8)	79 (3.1)	–	44 (2.4)	35 (4.8)
4+	57 (1.1)	49 (1.9)	–	32 (1.7)	17 (2.4)
Mean total length of hospital stays (95% CI)	9.25 (8.50‐10.00)	11.53 (10.36‐12.69)	.001	10.95 (9.71‐12.19)	12.67 (10.22‐15.13)

Abbreviation: CI, confidence interval.

^a^ Among 7838 patients with available healthcare utilization data at every visit.

Regarding clinical outcomes, Table S7 shows that DM had significant associations with the risk of atherosclerotic events (MI, stroke, and unstable angina requiring urgent revascularization) and deaths (both all‐cause and CV) but not with major bleeding events. Figure S2A,C show the cumulative incidence over 2 years for the composite of CV death, MI, or stroke by DM status; Figure S2B,D show the cumulative all‐cause mortality over 2 years by DM status. Patients with DM (especially insulin‐treated patients) had a significantly worse outcome for the composite endpoint and for all its components isolated (all‐cause mortality, MI, stroke, and unstable angina with urgent revascularization). Adjusted rate ratios for the composite of CV death, MI, or stroke were 1.55 (95% confidence interval [CI], 1.27‐1.89) for the comparison between DM and non‐DM, 1.41 (95% CI, 1.12‐1.78) for the comparison between noninsulin‐treated DM and non‐DM, and 1.83 (95% CI, 1.39‐2.39) for the comparison between insulin‐treated DM and non‐DM (Table 4). For all‐cause death, the corresponding rate ratios were 1.43 (95% CI, 1.13‐1.82), 1.31 (95% CI, 0.99‐1.72), and 1.66 (95% CI, 1.21‐2.28), respectively.

**TABLE 4:** Association of diabetes (overall and by insulin treatment) with the risk of CV death, MI, and stroke and the risk of all‐cause death over 2‐year follow‐up

	Crude RR (95% CI)	*P*‐value	Adjusted RR (95% CI)	*P*‐value
CV death, MI, and stroke				
Diabetes	1.85 (1.53‐2.25)	<.0001^a^	1.55 (1.27‐1.89)	<.0001^a^
Noninsulin‐treated diabetes	1.51 (1.21‐1.90)	<.0001^b^	1.41 (1.12‐1.78)	<.0001^b^
Insulin‐treated diabetes	2.70 (2.09‐3.48)		1.83 (1.39‐2.39)	
All‐cause death				
Diabetes	1.84 (1.46‐2.32)	<.0001^a^	1.43 (1.13‐1.82)^a^	.003
Noninsulin‐treated diabetes	1.45 (1.10‐1.90)	<.0001^b^	1.31 (0.99‐1.72)	.001^b^
Insulin‐treated diabetes	2.81 (2.09‐3.78)		1.66 (1.22‐2.28)	

*Note*: Results are from the Poisson regression model investigating the crude and adjusted association of diabetes with the rate of the composite outcome of CV death, MI, and stroke and the rate of all‐cause death, adjusted for variables in the TIGRIS risk index model: age ≥65 years, second prior MI, chronic kidney disease, heart failure, peripheral artery disease, CV event in the past 6 months, major bleed, medical management of index MI, diuretics, EQ‐5D‐weighted index score, region, and country as random effects.

Abbreviations: CI, confidence interval; CV, cardiovascular; EQ‐5D, EuroQol Research Foundation survey instrument for measuring self‐reported health status in 5 dimensions; MI, myocardial infarction; RR, rate ratio; TIGRIS, long‐Term rIsk, clinical manaGement and healthcare Resource utilization of stable coronary artery dISease.

^a^ Comparison between diabetes vs no diabetes.

^b^ Global test for no association with diabetes group (no diabetes, noninsulin‐treated diabetes, and insulin‐treated diabetes).

## 4 DISCUSSION

In our study, patients with DM and prior MI 1 to 3 years before enrollment were at a heightened risk of both MACE and all‐cause mortality compared with patients with prior MI and no DM. Moreover, there was a gradient in risk when patients with DM were analyzed according to insulin status, such that being on insulin was associated with an even higher risk for CV events. Patients with DM were also more frequently hospitalized and had more HRU, as expressed by the higher number of hospitalizations, emergency department visits, and outpatient visits. Finally, our study demonstrates that patient‐reported HRQoL, as measured by the EQ‐5D, was significantly associated with the presence of DM in those with prior MI.

Previous reports have suggested that patients with DM and no prior MI have the same risk of coronary death as patients with prior MI and no DM.^25^ Patients with both DM and prior MI comprised a subgroup of particularly high‐risk patients, with deaths due to coronary disease approaching 50% at 7 years.^25^ In the REACH registry, among patients with or at high risk for atherothrombotic CV disease, the presence of DM was associated with a nearly 50% increase in the risk of MACE, CV mortality, and all‐cause mortality during a 4‐year follow‐up.^5^ Comparable findings were published in a subanalysis from the PEGASUS‐TIMI 54 trial, a randomized study enrolling post‐MI patients whose inclusion criteria were similar to those in the TIGRIS registry. In PEGASUS‐TIMI 54, patients with DM also had an approximately 1.5‐fold increase in both MACE and all‐cause mortality compared with those without DM. Similar to our findings, there were also numerically higher rates of MACE and CV death in patients receiving insulin.^8^

DM is not only associated with incident CV disease and overall mortality but also negatively impacts HRQoL. In post‐ACS patients from the Veterans Health Administration, Rumsfeld et al demonstrated that DM had a negative impact on HRQoL that was comparable to the impact observed in depression and similar to the estimated HRQoL in other chronic diseases, such as HF, chronic obstructive pulmonary disease, and stroke.^12^ A publication examining long‐term MI survivors from the MONICA/KORA registry in Germany demonstrated that DM had a worse impact on EQ‐5D than either age ≥75 years or a history of more than one MI.^15^ Similarly, Shah et al showed that in patients with stable coronary artery disease (most of whom had a history of ACS), DM negatively affected all domains of the EQ‐5D, except self‐care.^26^ In contrast, a Canadian registry assessing HRQoL up to 1 year after MI found a positive association between DM and HRQoL scores measured by the 36‐item Short Form (SF‐36) questionnaire. The authors do acknowledge that this unexpected finding could be ascribed to uncontrolled confounders rendering a spurious association or to the fact that subjects lost to follow‐up in that cohort were more likely to have DM.^17^ These previous reports enrolled patients from single countries. Considering reports including patients from different global regions, a subanalysis from the randomized MERLIN trial also found that DM was associated with lower HRQoL scores in serial measurements for 1 year after ACS.^14^ In a subanalysis from the PLATO trial, DM was also independently associated with a worse HRQoL at 1 year after ACS, with parameter estimates comparable to those observed in patients having a prior MI.^16^ In contrast to these two reports, in the TIGRIS registry, patients had experienced their last MI more than 1 year ago; therefore, the HRQoL could be assessed more remotely from the acute event. In addition, owing to less stringent inclusion criteria than those required for randomized clinical trials, TIGRIS enrolled a population more representative of routine clinical practice in different economic and ethnic regions. Finally, it is important to note that some comorbidities that more often presented in the DM group may have influenced some of the EQ‐5D parameters. For example, obesity was more common in the DM group, which could have influenced mobility, usual activities, and pain. In addition, psychiatric diagnoses (including depression) were common among patients with diabetes and were related to a worse prognosis.^27^

Regarding HRU, few reports have explored this topic with high granularity in outpatients after MI. In a database derived from the United States health plans, employers, and government organizations, Menzin et al found that 21% of patients after ACS were hospitalized after 1 year and health‐related expenditures were mostly driven by hospitalizations. The presence of comorbidities (DM being the most common) was independently associated with a 50% increase in hospitalization.^28^ In a Swedish registry of post‐MI patients followed through a median of 6 years after the index event, Janzon et al demonstrated a marked increase in healthcare costs in the first year after MI. Subsequently, the costs remained consistent from the second year onward; those costs were mostly related to hospitalizations rather than medications and other healthcare resources. After the first year, in parallel with the population from our registry, there appeared to be an association of higher costs with the number of higher risk features (including DM).^18^ However, data regarding an association of costs and DM separately were not available in these two reports. In a cost‐effectiveness analysis from the TRITON‐TIMI 38 trial, in patients followed for up to 15 months after ACS treated with percutaneous coronary intervention for the index event, patients with DM had numerically higher costs compared with those without DM.^29^ Similarly, in a subanalysis from the PLATO trial, higher costs were associated with DM.^30^ Although we did not analyze direct costs, we assessed HRU in more detail than in prior studies. We have demonstrated that DM was associated with higher incidences of hospitalizations both in the 6 months prior to inclusion in the trial and in the 24 months of follow‐up, suggesting that DM contributes to a higher baseline cost use of hospital clinical resources, as well as exacerbating CV cost. Our results reinforce the recommendations of the WHO^31^ in terms of the importance of diet, physical activity, tobacco cessation, and lowering the levels of glucose and other known risk factors for atherosclerosis.

Our study has some limitations. First, although clinical endpoints were verified in medical records, they were not centrally adjudicated in the TIGRIS registry. It is reassuring that the increased risk of MACE and other clinically relevant events in patients with DM in the TIGRIS registry was consistent with that observed in the PEGASUS‐TIMI 54 trial, where all events were centrally adjudicated by a clinical events committee.^8^ Second, we ascertained HRQoL with only one type of instrument, the EQ‐5D. It should be mentioned that this questionnaire is widely used in HRQoL assessment outcome studies and has been validated in post‐MI patients; therefore, it is unlikely that results would be different should another instrument have been used.^32^ Third, we did not consider costs in our analysis of HRU. However, given that this is a global study across different types of healthcare systems and payers, it would be difficult to standardize costs in a meaningful manner. Considering that from similar publications the costs from hospitalizations dominate the health expenditures after MI,^18^ we believe our finding of an increase in hospitalizations (and length of stay[LoS]) and emergency visits among patients with DM is an adequate surrogate for healthcare‐related expenses. Fourth, owing to the specific inclusion criteria for the registry, DM, more than one prior MI, and renal dysfunction were enrichment risk factors. It is possible that this would affect the association of DM and risk factors, and our findings may not all be applicable to a lower risk post‐MI population. Finally, as with any observational study, we cannot establish a definitive causal relationship, since the results could be related to unmeasured confounders.

## 5 CONCLUSIONS

In stable outpatients' post‐MI, the presence of DM is associated with decreased long‐term survival and an increased risk for ischemic events. In addition, DM is associated with a higher HRU and worse HRQoL. These results could help inform healthcare system planning internationally in view of the global DM pandemic.

## AUTHOR CONTRIBUTIONS

José C. Nicolau, conception and design, analysis and interpretation of data, and drafting and writing the final version of the manuscript; Stuart J. Pocock and Ruth Owen, data analysis and interpretation and drafting and revision of the manuscript; Remo H.M. Furtado, data analysis and revision of the manuscript; David Brieger, Shaun G. Goodman, Mauricio G. Cohen, Tabassome Simon, Dirk Westermann, Christopher B. Granger, Richard Grieve, Satoshi Yasuda, Jiyan Chen, Katarina Hedman, Carl Mellström, and Gunnar Brandrup‐Wognsen, conduct of the registry and revision of the manuscript.

## Supporting information


**Appendix S1** Supporting information.

**Figure S1** Supporting information.

**Figure S2** Supporting information.

## Data Availability

Data underlying the findings described in this manuscript may be obtained in accordance with AstraZeneca's data sharing policy described at https://astrazenecagrouptrials.pharmacm.com/ST/Submission/Disclosure.
