# Supplementary material for: Diabetes association with self‐reported health, resource utilization, and prognosis post‐myocardial infarction
Source: Clin Cardiol. 2020 Nov 4;43(12):1352–61. doi: 10.1002/clc.23476 (PMC7724227; doi:10.1002/clc.23476)
Supplement: Supplementary file 1 — Appendix S1 Supporting information. [file CLC-43-1352-s001.docx]

**Supplementary File**

**TABLE S1.** Principal investigators for the TIGRIS Study (ClinicalTrials.gov: NCT01866904)

***Belgium***

M. Vrolix, C. Van Mieghem, M. Detollenaere, F. Cools, B. Vankelecom, D. Faes, S. Gevaert, P. Dejaegher

***Denmark***

H. Rickers, J. Skov Jensen, L. Holmvang, H. Nielsen, T. Bjerregaard Larsen, J. Brønnum-Schou, E. Vibeke Friis

***Finland***

J. Airaksinen, S. Vikman, J. Niva, A. Hadjikov

***France***

E. Ferrari, G. Range, G. Jean-Louis, O. Dubreuil, A. Ohanessian, T. Cuisset, P. Goube

***Germany***

P. Schwimmbeck, T. Schäufele, A. Joost, S. Lüders, E. Giannitsis, J. Brachmann, U. Zeymer, J. vom Dahl, R. Prondzinsky, O. Bruder, D. Westermann, T. Gori, F. Stahl, V. Rudolph, J. Weil, V. Wiechern, S. Behrens, B. Subin, D. Leistner, R. Gaub, S. Schellong, E. Schmidt, S. Markovic, H. Ebelt, J. Bott, C. Kadel, K. Kleinertz, H. Mudra, H. Darius, S. Achenbach, C. Contzen, J. Stößel, O. Maus, A. Rinke, C. Bickel

***Italy***

C. Cavallini, Z. Olivari, R. Mazzucco, G. Marenzi, S. Berti, P. Perrone Filardi, F. Pestelli, S. Novo, C. Rapezzi, C. Condorelli, G. Casolo, P. Franco Terrosu, C. Mauro

***Netherlands***

M.L.W.M. Wetering van de, F.F. Willems, G. Hoedemaker, B.J. Berg van den, E. Ronner, M.J.W. Götte, J.C.M. Hal van, Dr. Schellings, F.R. Hartog den, G.L. Bartels, J.F. Küpper, H.P. Beijerbacht, T. Lenderink, A.F.M. Kuijper, M.E.R.M. Daele van, N.Y.Y. Al-Windy, S.H.K. The

***Norway***

D. Nilsen, E.H. Øie, R.A.M. De Boer

***Portugal***

C. Gavina, L. Alves, P. Santos, J. Ferreira Santos, F. Matias, D. Ferreira, J. Mimoso, R. Lima, A. Gaspar

***Romania***

G. Stanciulescu, D. Darabantiu, D. Zdrenghea, N. Trocan, M. Dorobantu, M.C. Tomescu, S. Huidu, R. Musetescu, C. Bengus, M. Bogdan, S. Balanescu, L. Protopopescu, I. Manitiu, M. Vladoianu, L. Petrescu

***Spain***

J. Nevado Portero, G. Arquero, J. Sadurní Serrasolsas, J.A. Barrabes Riu, D. Pilar Mazon Ramos, V.I. Arrarte Esteban, A. Fernandez Ortiz, P. Pavia, J. Bruguera Cortada, A. Cequier Filiat, J.L. Blanco Coronado, F.J. Fernandez Portales, M. San Martin, R. Martin Asenjo, F. Marín Ortuño, M. Piqué, A. García Lledó, T. Ripoll, J.G. Sánchez Ramos

***Turkey***

E. Bozkurt, M. Bostan, E. Yeter, A. Çamsari, R. Altındağ, İ. Keleş, E. Atalar, C. Ceyhan

***United Kingdom***

A. Moriarty, R. Storey, B. O'Rourke, A. Pell, M.J. Dayer, R. Sharma, S. Hoole, A. Bakhai, J. Cotton

***Australia***

D. Brieger, N. Collins, M. Sader, C. Hammett, A. Lee, G. Nelson, P. Garrahy, P. Thompson, R. Jayasinghe, D. Eccleston, W. van Gaal, A. Whelan, J. French, J. Waites, R. Dick, K. Greaves

***China***

M. Genshan, X. Longgen, S. Guohai, C. Jiyan, G. Junbo, L. Bao, Y. Zhuhua, T. Guizhou, Q. Shubin, H. Yong, L. Han, J. Yan, Z. Yang, W. Yanni, Z. Zheng, C. Hongliang, Y. Lixia, Y. Zhenyu, Y. Jinchuan

***India***

R. Sivakumar, J.P. Sawhney, U. Kaul, V. Mehta, A. Abhyankar, R. Gupta, T. Nair, P.P. Mohanan, D. Kahali, H.K. Bali, P. Arora, M.S. Hirematth, A. Mullasari, P.C. Rath

***Japan***

Y. Morino, H. Niinuma, K. Nakao, T. Himi, S. Hokimoto, Y. Ozaki, T. Miura, S. Kitaguchi, K. Saku, J. Ako, W. Shimizu, K. Kawai, Y. Kobayashi, S. Mizuno, Y. Kazatani, K. Miwa, H. Shimokawa, N. Maejima, Y. Minoru, R. Kawaguchi, Y. Tomoguchi, T. Onodera, S. Hosokawa, T. Noguchi, S. Suwa, T. Matsumoto, T. Miyamoto, Y. Nakagawa, A. Wada, Y. Ikari, T. Ueno, K. Nishioka

***South Korea***

J. Yoon, K.S. Cha, T.-J. Cha, J.-K. Chae, C.-W. Nam, S.W. Kim, D.-I. Kim, S.M. Kim, M.-H. Jeong, J.S. Koh, J.-H. Lee, S.W. Choi

***Argentina***

J.L.C.N. Estrada, E.A. Duronto, R.B.S. Zarandon, E.A. Falu, M.S. Trivi, S.M. Macin, C.A. Rapallo, A.D. Hrabar, H.E. Fernandez, S.M. Muryan, D.J. Anauch, D.L. Paolantonio, O.A. Salomone

***Brazil***

A.P.M. Kormann, D.G. da Silva, Junior, J.C.F. Braga, L.N. Maia

***Canada***

C. Constance, S. Vizel, A. Glanz, A. Lamy, M. Gupta, Y. Pesant, W. Cantor, R. Petrella, R. Bessoudo (Marr), A.S. Pandey, A. Bell, I. Teitelbaum, R. Chehayeb, R. Dupuis

***Colombia***

C. Arana, M. Herrera, D.I. Molina, M. Urina, N. Jaramillo, R. Fernandez, B. Vesga, H. Duque, J.L. Accini, F. Manzur, C. Jaramillo

***Mexico***

E.D. y Díaz, A.B. Ruiz, G.R. Martínez, I.R. Briones, F.P. Padilla, J.C.P. Alva, O.F. Fierro

***United States of America***

J.J. Kmetzo, N. Mayer, N. Singh, R. Sangrigoli, W. Bennett, D. Schneider, E. Claxton, M. Newell, N.K. Mann, P. Gordon, E. Korban, R. Mehta, M. McKenzie, K. Friedman, W.J. French, T. Wang, W. Sheldon, J.K. Amin, M. Baig, T. Haddad, A. Wiseman, B. Herbstman, H. Taheri, L. Blacher, G. Stouffer, K. Tummalapalli, J. Ghitelman, A. Schwarcz, R. D'Agostino, R. Davidson, J. Duffy (Tonkon), E. Kaluski, J. Petersen II, M. Ball, W. Batchelor

***Venezuela***

N. Antepara, S. Tovar, L. Fariña, C. Delgado, Á. Avendaño

**TABLE S2** Baseline characteristics of the population by diabetes status

|  | **No diabetes** | **Diabetes** | ***P*-value** |
| --- | --- | --- | --- |
|  | **N=6009** | **N=2959** |  |
| **Female** | 1368±22.8 | 780±26.4 | <.001 |
| **Age (years), mean±SD** | 67.5±8.6 | 65.8±8.6 | <.0001 |
| **BMI (kg/m^2^), mean±SD** | 26.87±4.34 | 28.25±5.18 | <.0001 |
| **SBP (mmHg), mean±SD** | 131.11±17.47 | 132.51±18.15 | <.001 |
| **DBP (mmHg), mean±SD** | 76.81±10.36 | 76.20±10.53 | .01 |
| **Heart rate (bpm), mean±SD** | 67.33±10.52 | 70.50±10.59 | <.0001 |
| **Smoking status** |  |  |  |
| Never | 2154 (35.9) | 1219 (41.2) | <.0001 |
| Former | 2999 (49.9) | 1351 (45.7) |  |
| Current | 855 (14.2) | 388 (13.1) |  |
| **Second prior MI** | 579 (9.6) | 3439 (11.5) | .007 |
| **Multivessel disease** | 4109 (68.4) | 1798 (60.8) | <.0001 |
| **Chronic kidney disease** | 383 (6.4) | 304 (10.3) | <.0001 |
| **Peripheral artery disease** | 310 (5.2) | 289 (9.8) | <.0001 |
| **Congestive heart failure** | 598 (10.0) | 431 (14.6) | <.0001 |
| **Chronic anemia** | 136 (2.3) | 121 (4.1) | <.0001 |
| **Angina** | 535 (8.9) | 361 (12.2) | <.0001 |
| **Atrial fibrillation** | 476 (7.9) | 248 (8.4) | .45 |
| **Permanent pacemaker** | 127 (2.1) | 73 (2.5) | .29 |
| **Implantable cardioverter defibrillator** | 114 (1.9) | 79 (2.7) | .02 |
| **Stroke** | 225 (3.7) | 174 (5.9) | <.0001 |
| **TIA** | 132 (2.2) | 60 (2.0) | .60 |
| **Venous thromboembolism** | 100 (1.7) | 49 (1.7) | .98 |
| **Cancer** | 412 (6.9) | 196 (6.6) | .68 |
| **COPD** | 413 (6.9) | 230 (7.8) | .12 |
| **Hospitalization in prior 6 months for CV or bleeding event** | 298 (5.0) | 188 (6.4) | .006 |
| **Time since index MI (months)** |  |  |  |
| 12-18 | 2025 (33.7) | 1029 (34.8) | .63 |
| 18-24 | 1503 (25.0) | 737 (24.9) |  |
| 24-30 | 1364 (22.7) | 672 (22.7) |  |
| 30-36 | 1117 (18.6) | 521 (17.6) |  |
| **Type of index MI** |  |  |  |
| STEMI | 3234 (53.8) | 1462 (49.4) | <.0001 |
| NSTEMI | 2456 (40.9) | 1284 (43.4) |  |
| Unknown | 319 (5.3) | 213 (7.2) |  |
| **Management of index MI** |  |  |  |
| PCI | 4922 (81.9) | 2317 (78.3) | <.0001 |
| CABG | 433 (7.2) | 227 (7.7) |  |
| Medical | 654 (10.9) | 415 (14.0) |  |
| **Diuretics** | 1323 (22.4) | 924 (31.6) | <.0001 |
| **Antidepressants** | 378 (6.4) | 248 (8.5) | <.001 |

Summary statistics are given as n (%) unless stated otherwise.

Abbreviations: BMI, body mass index; bpm, beats per minute; CABG, coronary artery bypass grafting; COPD, chronic obstructive pulmonary disease; CV, cardiovascular; DBP, diastolic blood pressure; MI, myocardial infarction; NSTEMI, non–ST-elevation myocardial infarction; PCI, percutaneous coronary intervention; SBP, systolic blood pressure; SD, standard deviation; STEMI, ST-elevation myocardial infarction; TIA, transient ischemic attack.

# TABLE S3 Baseline characteristics of the diabetes population by insulin use

|  | **Non–insulin-treated diabetes** | **Insulin-treated diabetes** | ***P*-value** |
| --- | --- | --- | --- |
|  | **N=2090** | **N=869** |  |
| **Female** | 508±24.3 | 272±31.3 | <.0001 |
| **Age (years), mean±SD** | 65.9±8.5 | 65.43±8.6 | .16 |
| **BMI (kg/m^2^), mean±SD** | 27.98±4.88 | 28.88±5.79 | .0001 |
| **SBP (mmHg), mean±SD** | 132.28±17.90 | 133.08±18.75 | .29 |
| **DBP (mmHg), mean±SD** | 76.48±10.45 | 75.53±10.72 | .03 |
| **Heart rate (bpm), mean±SD** | 70.15±10.59 | 71.33±10.57 | .007 |
| **Smoking status** |  |  | <.0001 |
| Never | 802 (38.4) | 417 (48.0) |  |
| Former | 987 (47.2) | 364 (41.9) |  |
| Current | 300 (14.4) | 88 (10.1) |  |
| **Second prior MI** | 218 (10.5) | 121 (13.9) | .007 |
| **Multivessel disease** | 1247 (59.7) | 551 (63.4) | .06 |
| **Chronic kidney disease** | 181 (8.7) | 123 (14.2) | <.0001 |
| **Peripheral artery disease** | 166 (7.9) | 123 (14.2) | <.0001 |
| **Congestive heart failure** | 268 (12.8) | 163 (18.8) | <.0001 |
| **Chronic anemia** | 63 (3.0) | 58 (6.7) | <.0001 |
| **Angina** | 244 (11.7) | 117 (13.8) | .18 |
| **Atrial fibrillation** | 163 (7.8) | 85 (9.8) | .08 |
| **Permanent pacemaker** | 46 (2.2) | 27 (3.1) | .15 |
| **Implantable cardioverter defibrillator** | 44 (2.1) | 35 (4.0) | .003 |
| **Stroke** | 112 (5.4) | 62 (7.1) | .06 |
| **TIA** | 39 (1.9) | 21 (2.4) | .33 |
| **Venous thromboembolism** | 33 (1.6) | 16 (1.8) | .61 |
| **Cancer** | 137 (6.6) | 59 (6.8) | .82 |
| **COPD** | 159 (7.6) | 71 (8.2) | .60 |
| **Hospitalization in prior 6 months for CV or bleeding event** | 121 (5.8) | 67 (7.7) | .05 |
| **Time since index MI (months)** |  |  | .85 |
| 12-18 | 711 (34.0) | 318 (36.6) |  |
| 18-24 | 541 (25.9) | 196 (22.6) |  |
| 24-30 | 476 (22.8) | 196 (22.6) |  |
| 30-36 | 362 (17.3) | 159 (18.3) |  |
| **Type of index MI** |  |  | .10 |
| STEMI | 1057 (50.6) | 405 (46.6) |  |
| NSTEMI | 881 (42.2) | 403 (46.4) |  |
| Unknown | 152 (7.3) | 61 (7.0) |  |
| **Management of index MI** |  |  | .001 |
| PCI | 1669 (79.9) | 648 (74.6) |  |
| CABG | 139 (6.7) | 88 (10.1) |  |
| Medical | 282 (13.5) | 133 (15.3) |  |
| **Diuretics** | 585 (28.3) | 339 (39.4) | <.0001 |
| **Antidepressants** | 144 (7.0) | 104 (12.3) | <.0001 |

Summary statistics are given as n (%) unless otherwise stated.

Abbreviations: BMI, body mass index; bpm, beats per minute; CABG, coronary artery bypass grafting; COPD, chronic obstructive pulmonary disease; CV, cardiovascular; DBP, diastolic blood pressure; MI, myocardial infarction; NSTEMI, non–ST-elevation myocardial infarction; PCI, percutaneous coronary intervention; SBP, systolic blood pressure; SD, standard deviation; STEMI, ST-elevation myocardial infarction; TIA, transient ischemic attack.

# TABLE S4 Region and country by diabetes status

|  | **Number of patients, n** | **Patients with diabetes, n (%)** |
| --- | --- | --- |
|  | **N=8968** | **N=2959** |
| **Region** |  |  |
| Europe | 4110 | 1201 (29.2) |
| Asia and Australia | 2774 | 1026 (37.0) |
| North America | 980 | 331 (33.8) |
| Latin America | 1104 | 401 (36.3) |
| **Country** |  |  |
| Germany | 1042 | 323 (31.0) |
| Spain | 619 | 261 (42.2) |
| Netherlands | 591 | 113 (19.1) |
| Italy | 391 | 98 (25.1) |
| Romania | 343 | 129 (37.6) |
| Denmark | 300 | 47 (15.7) |
| UK | 265 | 54 (20.4) |
| Belgium | 140 | 43 (30.7) |
| Portugal | 91 | 37 (40.7) |
| Finland | 72 | 12 (16.7) |
| Norway | 46 | 10 (21.7) |
| France | 37 | 8 (21.6) |
| Japan | 682 | 296 (43.4) |
| China | 747 | 243 (32.5) |
| India | 493 | 251 (50.9) |
| South Korea | 453 | 144 (31.8) |
| Australia | 399 | 99 (24.8) |
| Turkey | 173 | 66 (38.2) |
| USA | 735 | 264 (35.9) |
| Canada | 245 | 67 (27.4) |
| Argentina | 451 | 134 (29.7) |
| Colombia | 348 | 124 (35.6) |
| Brazil | 115 | 63 (54.8) |
| Venezuela | 104 | 38 (36.5) |
| Mexico | 86 | 42 (48.8) |

Summary statistics are given as n (%).

Abbreviations: UK, United Kingdom; USA, United States of America.

# TABLE S5 Evidence-based treatments at enrolment by diabetes status

|  | **No diabetes** | **Diabetes** | ***P*-value** | **Non–insulin-treated diabetes** | **Insulin-treated diabetes** |
| --- | --- | --- | --- | --- | --- |
|  | **N=6009** | **N=2959** |  | **N=2090** | **N=869** |
| **Antithrombotic drugs**^a^ |  |  |  |  |  |
| None | 103 (1.7) | 77 (2.6) | <.0001 | 54 (2.6) | 23 (2.7) |
| SAPT | 4040 (67.4) | 1826 (61.8) |  | 1279 (61.3) | 547 (63.1) |
| DAPT | 1457 (24.3) | 826 (28.0) |  | 615 (29.5) | 211 (24.3) |
| Anticoagulant | 395 (6.6) | 224 (7.6) |  | 138 (6.6) | 86 (9.9) |
| **ACE inhibitor or ARB** | 4358 (73.6) | 2306 (78.8) | <.0001 | 1635 (79.1) | 671 (78.1) |
| **Beta-blocker** | 4696 (79.2) | 2386 (81.5) | .01 | 1674 (80.9) | 712 (83.0) |
| **Statin or other lipid‑lowering drug** | 5617 (94.7) | 2730 (93.3) | .008 | 1924 (93.1) | 806 (93.8) |

Summary statistics are given as n (%).

Abbreviations: ACE, angiotensin converting enzyme; ARB, angiotensin receptor blocker; DAPT, dual antiplatelet therapy; SAPT, single antiplatelet therapy.

^a^Patients taking anticoagulants were categorized as AC regardless of their antiplatelet use.

# TABLE S6 Healthcare utilization over 2-year follow-up by diabetes status^a^

|  | **No diabetes** | **Diabetes** | ***P*-value** | **Non–insulin-treated diabetes** | **Insulin-treated diabetes** |
| --- | --- | --- | --- | --- | --- |
|  | **N=5249** | **N=2589** |  | **N=1866** | **N=723** |
| **CV hospitalizations** | | | | | |
| **Mean number of hospitalizations (95% CI)** | 0.16 (0.14-0.17) | 0.23 (0.21-0.26) |  | 0.21 (0.18-0.23) | 0.30 (0.25-0.35) |
| **Number of hospitalizations, N (%)** |  |  |  |  |  |
| 0 | 4618 (88.0) | 2155 (83.2) | <.0001 | 1575 (84.4) | 580 (80.2) |
| 1 | 486 (9.3) | 318 (12.3) |  | 225 (12.1) | 93 (12.9) |
| 2 | 116 (2.2) | 82 (3.2) |  | 47 (2.5) | 35 (4.8) |
| 3 | 17 (0.3) | 23 (0.9) |  | 13 (0.7) | 10 (1.4) |
| 4+ | 12 (0.2) | 11 (0.4) |  | 6 (0.3) | 5 (0.7) |
| **Mean total length of hospital stay (95% CI)** | 6.7 (6.0-7.3) | 8.7 (7.6-9.9) | .002 | 8.1 (6.9-9.3) | 10.1 (7.7-12.4) |
| **Bleeding hospitalizations** | | | | | |
| **Mean number of hospitalizations (95% CI)** | 0.02 (0.01-0.02) | 0.02 (0.01-0.02) |  | 0.01 (0.01-0.02) | 0.03 (0.02-0.04) |
| **Number of hospitalizations, N (%)** |  |  |  |  |  |
| 0 | 5160 (98.3) | 2546 (98.3) | .99 | 1841 (98.7) | 705 (97.5) |
| 1 | 83 (1.6) | 39 (1.5) |  | 24 (1.3) | 15 (2.1) |
| 2+ | 6 (0.1) | 4 (0.2) |  | 1 (0.1) | 3 (0.4) |
| **Mean total length of hospital stay (95% CI)** | 7.0 (5.3-8.7) | 6.3 (4.6-8.1) | .60 | 5.6 (4.0-7.2) | 7.3 (3.7-11.0) |
| **ER visits for a CV or bleeding event** | | | | | |
| **Mean number of ER visits (95% CI)** | 0.12 (0.10-0.13) | 0.14 (0.12-0.17) |  | 0.13 (0.11-0.16) | 0.17 (0.13-0.21) |
| **Number of ER visits, N (%)** |  |  |  |  |  |
| 0 | 4788 (91.2) | 2335 (90.2) | .02 | 1692 (90.7) | 643 (88.9) |
| 1 | 372 (7.1) | 188 (7.3) |  | 134 (7.2) | 54 (7.5) |
| 2 | 63 (1.2) | 39 (1.5) |  | 23 (1.2) | 16 (2.2) |
| 3+ | 26 (0.5) | 27 (1.0) |  | 17 (0.9) | 10 (1.4) |
| **GP visits for a CV or bleeding event** | | | | | |
| **Mean number of GP visits (95% CI)** | 4.32 (4.13-4.51) | 4.89 (4.58-5.21) |  | 4.81 (4.44-5.18) | 5.10 (4.48-5.71) |
| **Number of GP visits, N (%)** |  |  |  |  |  |
| 0 | 2141 (40.8) | 1070 (41.3) | .02 | 812 (43.5) | 258 (35.7) |
| 1-4 | 1538 (29.3) | 687 (26.5) |  | 480 (25.7) | 207 (28.6) |
| 5-9 | 783 (14.9) | 362 (14.0) |  | 239 (12.8) | 123 (17.0) |
| 10-19 | 548 (10.4) | 307 (11.9) |  | 207 (11.1) | 100 (13.8) |
| 20-29 | 171 (3.3) | 123 (4.8) |  | 97 (5.2) | 26 (3.6) |
| 30+ | 68 (1.3) | 40 (1.5) |  | 31 (1.7) | 9 (1.2) |
| **Cardiologist visits** | | | | | |
| **Mean number of visits to cardiologist (95% CI)** | 4.01 (3.84-4.17) | 4.84 (4.54-5.14) |  | 4.94 (4.59-5.29) | 4.59 (4.02-5.17) |
| **Number of visits to cardiologist, N (%)** |  |  |  |  |  |
| 0 | 1374 (26.2) | 648 (25.0) | <.0001 | 491 (26.3) | 157 (21.7) |
| 1-4 | 25548 (48.5) | 1180 (45.6) |  | 802 (43.0) | 378 (52.3) |
| 5-9 | 745 (14.2) | 383 (14.8) |  | 283 (15.2) | 100 (13.8) |
| 10-19 | 391 (7.5) | 238 (9.2) |  | 185 (9.9) | 53 (7.3) |
| 20-29 | 132 (2.5) | 103 (4.0) |  | 76 (4.1) | 27 (3.7) |
| 30+ | 59 (1.1) | 37 (1.4) |  | 29 (1.6) | 8 (1.1) |
| **Other specialist visits** | | | | | |
| **Mean number of visits to other specialists (95% CI)** | 1.47 (1.37-1.57) | 2.03 (1.86-2.21) |  | 1.76 (1.57-1.94) | 2.75 (2.33-3.17) |
| **Number of visits to other specialists, N (%)** |  |  |  |  |  |
| 0 | 3257 (62.1) | 1520 (58.7) | <.0001 | 1137 (60.9) | 383 (53.0) |
| 1-4 | 1495 (28.5) | 713 (27.5) |  | 505 (27.1) | 208 (28.8) |
| 5-9 | 325 (6.2) | 206 (8.0) |  | 134 (7.2) | 72 (10.0) |
| 10-19 | 133 (2.5) | 115 (4.4) |  | 72 (3.9) | 43 (6.0) |
| 20-29 | 29 (0.6) | 25 (1.0) |  | 13 (0.7) | 12 (1.7) |
| 30+ | 10 (0.2) | 10 (0.4) |  | 5 (0.3) | 5 (0.7) |

Abbreviations: CI, confidence interval; CV, cardiovascular; ER, emergency room; GP, general practitioner.

^a^Among 7838 patients with available healthcare utilization data at every visit and includes events from all hospitalizations/visits.

# TABLE S7 Clinical outcomes by diabetes status

|  | **No diabetes**  **N=6009** | **Diabetes**  **N=2959** | ***P*-value** | **Non–insulin-treated diabetes**  **N=2090** | **Insulin-treated diabetes**  **N=869** |
| --- | --- | --- | --- | --- | --- |
| **Primary composite^a^** | 341 (5.7) | 275 (9.3) | <.0001 | 159 (7.6) | 116 (13.3) |
| **All-cause death** | 154 (2.6) | 138 (4.7) | <.0001 | 77 (3.7) | 61 (7.0) |
| **MI** | 113 (1.9) | 82 (2.8) | .007 | 46 (2.2) | 36 (4.1) |
| **Stroke** | 33 (0.5) | 24 (0.8) | <.0001 | 15 (0.7) | 9 (1.0) |
| **Unstable angina with urgent revascularization** | 54 (0.9) | 47 (1.6) | .004 | 30 (1.4) | 17 (2.0) |
| **CV death** | 77 (1.3) | 98 (3.3) | <.0001 | 56 (2.7) | 42 (4.9) |
| **Composite of CV death, MI, and stroke** | 219 (3.6) | 196 (6.6) | <.0001 | 114 (5.5) | 82 (9.4) |
| **Major bleeding event** | 76 (1.3) | 33 (1.1) | .54 | 16 (0.8) | 17 (2.0) |

Summary statistics are given as n (%); include only the first event.

Abbreviations: CV, cardiovascular; MI, myocardial infarction.

^a^Composite of all-cause death, MI, stroke, and unstable angina with urgent revascularization.

#
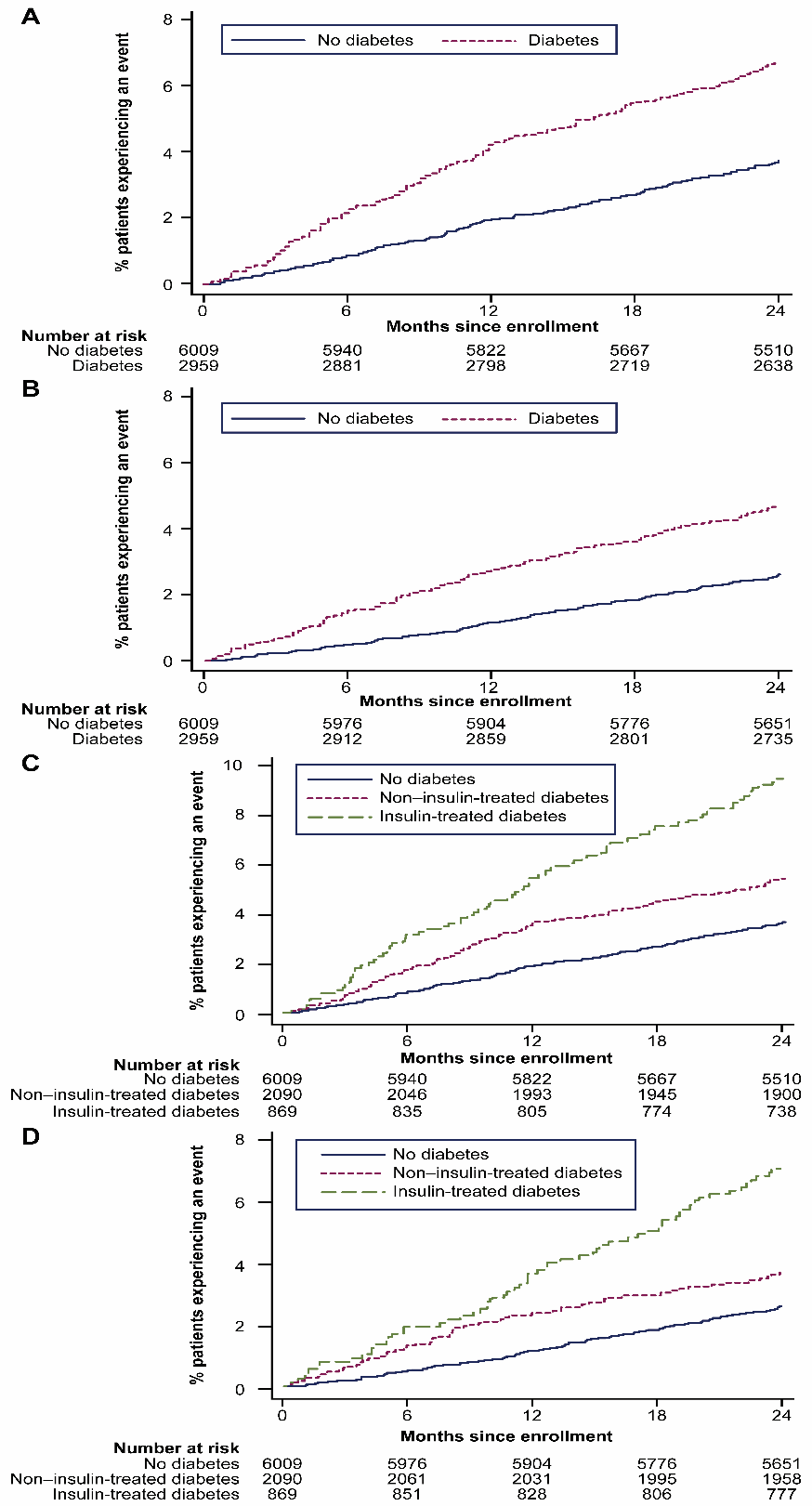


# FIGURE S1 Kaplan-Meier plot of time to (A) the composite endpoint of CV death, MI, and stroke by diabetes status; (B) all-cause death by diabetes status; (C) the composite endpoint of CV death, MI, and stroke by diabetes status and treatment by diabetes status and treatment with insulin; and (D) all-cause death by diabetes status and treatment by diabetes status and treatment with insulin.

Abbreviations: CV, cardiovascular; MI, myocardial infarction.
