# Supplementary figures and images for: Diabetes association with self‐reported health, resource utilization, and prognosis post‐myocardial infarction
Source: Clin Cardiol. 2020 Nov 4;43(12):1352–61. doi: 10.1002/clc.23476 (PMC7724227; doi:10.1002/clc.23476)

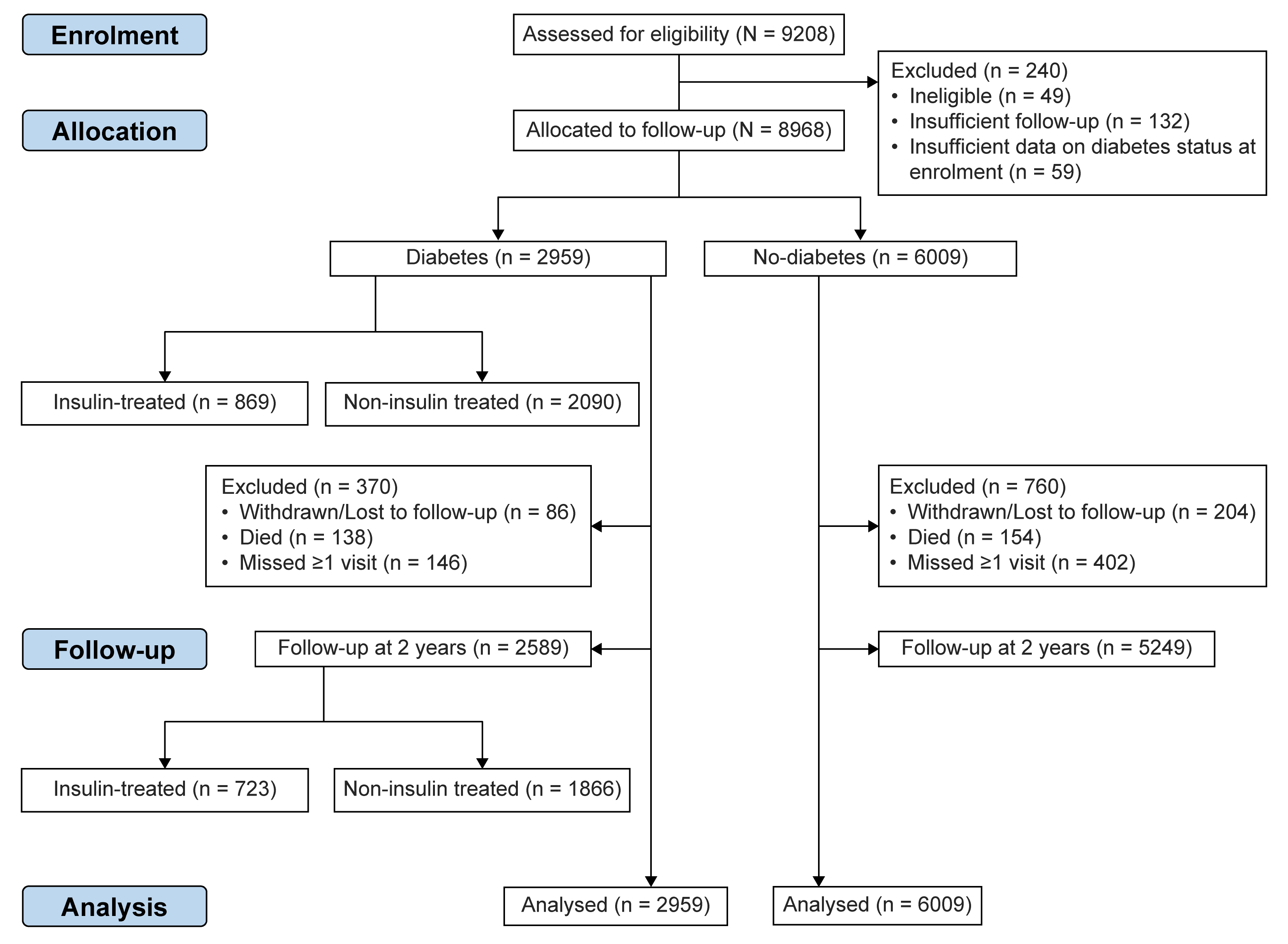

Supplement: Supplementary file 2 — Figure S1 Supporting information. [file CLC-43-1352-s002.tif]
